# Advantages of micro‐CT in the case of a complex dismemberment

**DOI:** 10.1111/1556-4029.15007

**Published:** 2022-02-04

**Authors:** Kirsty Alsop, Danielle G. Norman, Waltraud Baier, Jim Colclough, Mark A. Williams

**Affiliations:** ^1^ Forensic Centre for Digital Scanning and 3D Printing, WMG University of Warwick Coventry UK; ^2^ West Midlands Police Birmingham UK

**Keywords:** case report, dismemberment, false start, micro‐CT, tool marks

## Abstract

This case study reports the advantages of micro‐CT to aid the investigative process in a complex dismemberment case. Micro‐CT was successfully implemented to scan all skeletal remains of a dismembered female. The digital models were utilized to (i) screen for any further injuries not related to the dismemberment, (ii) provide measurements from false starts non‐destructively, and (iii) visually represent the evidence in a structured format in court to improve the understanding of the forensic evidence by the jury. Acquiring high‐resolution scans in this manner improved the efficiency of the forensic investigation by screening the remains and provided complementary toolmark evidence to the investigating team and forensic pathologist. A total of 14 false starts were identified along with the directionality of each dismemberment cut. Furthermore, the visual 3D representation of the remains in court provided a powerful tool to communicate this important evidence to the jury and form a prosecution narrative. As a forensic radiological method, micro‐CT provided valuable information both in the investigation and the court presentation.

**Highlights**

- Micro‐CT was used to aid in a complex case of dismemberment.
- Digital models were created to screen for further injuries without maceration.
- Measurements on 14 false starts were provided non‐destructively.
- A visual court presentation was produced as a powerful tool to communicate the evidence.

## 1 INTRODUCTION

Dismemberment cases, though not common in the UK, provide significant challenges to the investigative process and require substantial resources. Separation of a body increases the complexity of disposal site(s) and likelihood of missing body parts [1]. Nevertheless, due to the inherent visceral nature of the dismemberment process, the probability of finding evidence at the crime scenes involved is high [1]. The challenge lies in collating the evidence from a wealth of forensic experts and utilizing all available resources during the police investigation. The case presented provided more complex challenges than others seen at the same institution due to the 10 dismemberment sites across the body and the necessity to micro‐CT scan the entirety of the remains [2].

Forensic investigation of a dismembered body typically employs the expertise of a forensic anthropologist or toolmark specialist [3]. However, with the advancement of non‐invasive imaging modalities, such as radiological techniques like computed tomography (CT) [4, 5, 6], evidence can also be digitally captured prior to any further applications. While radiography and “medical” grade CT scans can be applied with great success [7, 8, 9], these methodologies can obscure important detail below their typical scanning resolution of approximately 600 μm [10, 11]. The advancement of micro‐CT for forensic applications, with a spatial resolution of 500 nm −120 μm [12], allows for more minutiae to be digitally captured in 3D [6] which is important for toolmark and fracture examination and can be applied complementarily to traditional toolmark analyses.

Micro‐CT has proven to be useful in forensic research in the areas of blunt force injuries [13], sharp force injuries [14, 15, 16], false start analysis [17, 18, 19, 20], and ballistic analysis [21, 22, 23]. Case reports featuring this technology as part of live police investigations are limited with few notable exceptions in Baier and colleagues' work [24, 25] on dismemberment and rib fractures, respectively. This case report details how micro‐CT can assist a complex police dismemberment case by (i) providing a complementary technique to physical maceration, (ii) identifying and characterizing false starts non‐invasively, and (iii) visually presenting the evidence to a jury.

## 2 CASE BACKGROUND

Following the disappearance of a female in the UK the investigation began to identify her whereabouts as a missing person. A male suspect was quickly identified as being in the company of the missing person shortly before her disappearance and arrested on suspicion of kidnapping. On arrest, it was believed that the suspect had in fact murdered the missing female. Investigation into the suspect identified the purchase of a new carpet and underlay the day after the victim went missing. A forensic examination of the suspect’s residence found staining under the area of new carpet which tested positive for the presence of blood, which was later identified through DNA as belonging to the victim. The suspect and their partner were later arrested for murder.

Police search teams recovered human remains located at two deposition sites, positioned approximately 25 m apart. The suspected body parts were wrapped in black plastic and appeared to have been damaged by fire and possible animal interference. Post‐mortem CT scans and a preliminary post‐mortem were performed at the local hospital which noted that the left leg, thorax, and left foot were missing from the recovered samples. These remaining body parts were located approximately 2 weeks later close to the site where the original remains were discovered. The remaining body parts were also CT scanned, and a second post‐mortem was conducted. All body parts containing skeletal elements were then submitted for micro‐CT scanning at the authors' institution.

Due to the established partnership between the authors' institution and the police, micro‐CT is frequently implemented in homicide cases in the UK and has been applied to over 200 cases nationwide. Importantly, this partnership allows access to micro‐CT to all UK police forces at a reasonable cost. The nature of forensic services in the UK demands external forensic analysis for fields such as histopathology, toolmark examination, and, in this case, micro‐CT. No great demand is placed on the police force to implement micro‐CT but is now considered standard practice in many homicide investigations.

## 3 MATERIALS AND METHODS

**FIGURE 1:**
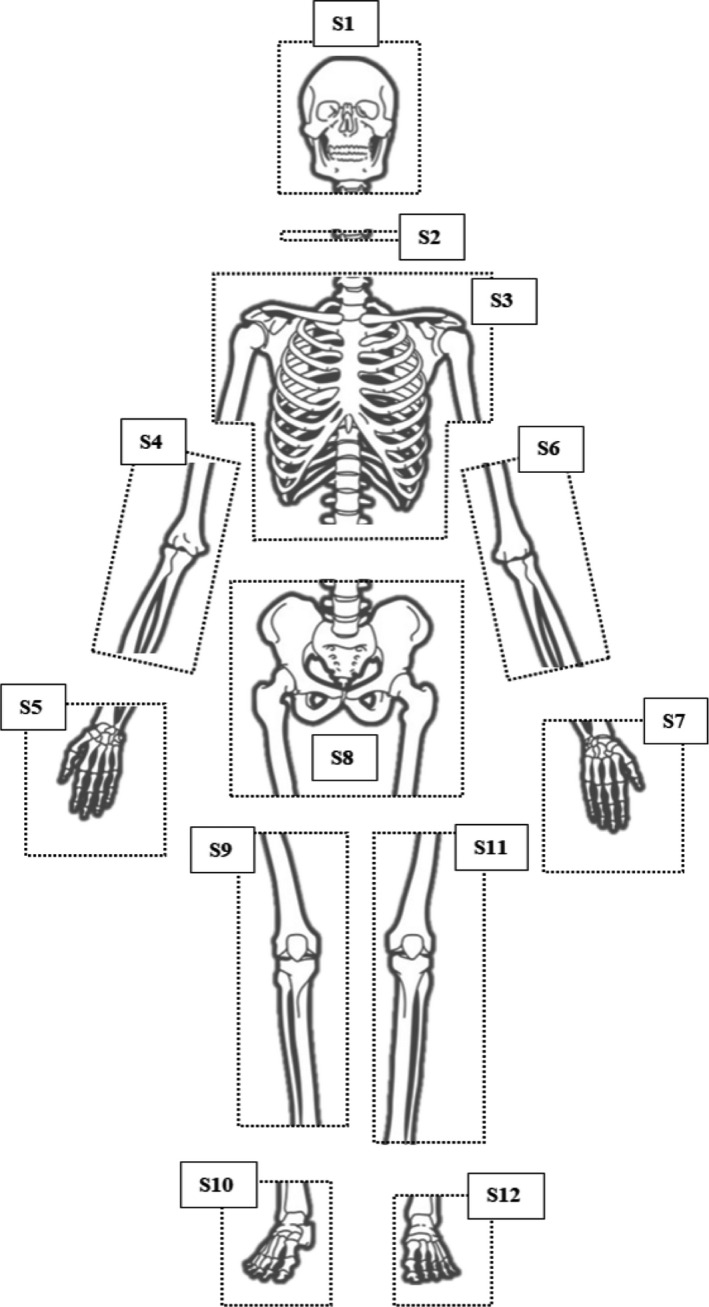
Diagram of the approximate locations of the dismemberment sites and sample IDs

During the dismemberment process, the body was sectioned into multiple fragments, 12 of which contained skeletal elements. Each of the 12 samples were packaged in sealed plastic containers by the police and hospital mortuary team following the post‐mortem examination. Table 1 and Figure 1 detail the samples submitted for high‐resolution micro‐CT imaging at the authors' institution. During the post‐mortem, no further segmentation was performed by the pathologist; therefore, the application of micro‐CT was completely non‐intrusive. This process allowed digital preservation of the samples prior to any further analysis. The post‐processing of the scans included the production of animations and presentation.

**TABLE 1:** Sample ID, skeletal element(s), and micro‐CT scanner used for each sample submitted for scanning

Sample ID	Skeletal element(s)	Micro‐CT scanner used	Voxel resolution
S1	Skull & cervical vertebrae	Nikon XT H 225/320LC	58 μm
S2	Section of C5 vertebra	Nikon XT H 225/320LC	45 μm
S3	Torso with proximal left and right humerii	Nikon 450/750 RT	79 μm
S4	Right arm (mid‐humerus to distal radius/ulna)	Nikon XT H 225/320LC	56 μm
S5	Right hand and distal radius & ulna	Nikon XT H 225/320LC	56 μm
S6	Left arm (mid‐humerus to distal radius/ulna)	Nikon XT H 225/320LC	51 μm
S7	Left hand and distal radius & ulna	Nikon XT H 225/320LC	58 μm
S8	Pelvis with proximal left and right femora	Nikon 450/750 RT	82 μm
S9	Right leg (mid‐femur to distal tibia/fibula)	Nikon XT H 225/320LC	53 μm
S10	Right foot and distal tibia/fibula	Nikon XT H 225/320LC	58 μm
S11	Left leg (mid‐femur to distal tibia/fibula)	Nikon XT H 225/320LC	51 μm
S12	Left foot and distal tibia/fibula	Nikon XT H 225/320LC	49 μm

Each sample was scanned using either a Nikon XT H 225/320LC or Nikon 450/750 RT depending on container diameter. Scan parameters were adjusted appropriately for each sample to ensure optimal image contrast, clarity, and field of view. The resulting scan parameters were as follows: power between 15.5 and 30 W, voltage between 100 and140 kV, and exposure between 0.25 and 1 s resulting in voxel resolutions of between 48 and 126 μm. The 3D micro‐CT data were reconstructed from the 3142 X‐ray projections using Nikon’s proprietary software, *CT Pro,* and then exported to VGStudio MAX 2.2 (VolumeGraphics, Heidelberg, Germany). A calibrated workpiece was included in each scan to allow voxel rescaling and improve measurement accuracy [26, 27].

VGStudio MAX 2.2 was utilized to digitally filter the soft tissue to view the 3D reconstruction of each bone (digital maceration), cataloguing of the injuries, examine any toolmarks on the bones, and generate 3D videos for court presentation. The digital filter used an ISO50 peak‐to‐peak threshold which is the default option in VGStudio Max—the results of this were manually inspected. The location of dismemberment cuts and other tool mark features, including scratches, false starts, and breakaway spurs, were reported visually to show the full extent of the dismemberment trauma.

Following production of a forensic report for the investigating team and pathologist, a presentation was produced for the court trial. Communicating the evidence to a jury is an integral part of the forensic process, however, can be challenging both technically and ethically particularly with pathological evidence. The digital micro‐CT scans of the samples were visually represented as greyscale 3‐dimensional digital models which rotated around an axis as a video. A court presentation was produced to show how dismemberment sites on corresponding samples visually correlate and to highlight the false starts, cut sites, and breakaway spurs.

Production of the micro‐CT scans, dismemberment analyses, measurements, and court presentation were conducted by micro‐CT experts with qualifications and experience with forensic anthropology. All results were passed to the forensic pathologist for analysis of trauma.

## 4 RESULTS

Each sample was digitally captured through the non‐invasive micro‐CT method outlined above. The high resolution and digital filtration successfully allowed the skeletal elements to be viewed in 3D without extreme disruption to the body. This method identified all areas of trauma on the bone and, following examination by the forensic pathologist, ruled out any further skeletal trauma and avoided the need for physical maceration in this case.

A total of 14 measurable false starts were identified close to the dismemberment ends. Micro‐CT allowed for the visualization of the false starts along with breakaway spurs and other features. Table 2 shows the location and width of the identified false starts.

**TABLE 2:** False start ID, location, and measured width (mm) of each false start

False start ID	Location	Width (mm)
S1.1	Left posterolateral C5 vertebra	1.08–1.24
S3.2	Lateral right humerus	1.17–1.68
S4.3	Lateral right radius	0.93
S4.4	Medial right ulna	0.62
S6.5	Posterior left radius – 4 mm from cut end	1.03
S6.6	Posterior left radius – 1 mm from cut end	1.00
S6.7	Medial left radius – 3 mm from cut end	1.05
S6.8	Medial left radius – 2 mm from cut end	0.91
S7.9	Medial left ulna	1.10
S8.10	Lateral right femur	1.43
S8.11	Lateral left femur – 9 mm from cut end	1.02
S8.12	Lateral left femur – 4 mm from cut end	1.14
S8.13	Lateral left femur – 1 mm from cut end	0.89
S11.14	Medial left tibia	1.00

**FIGURE 2:**
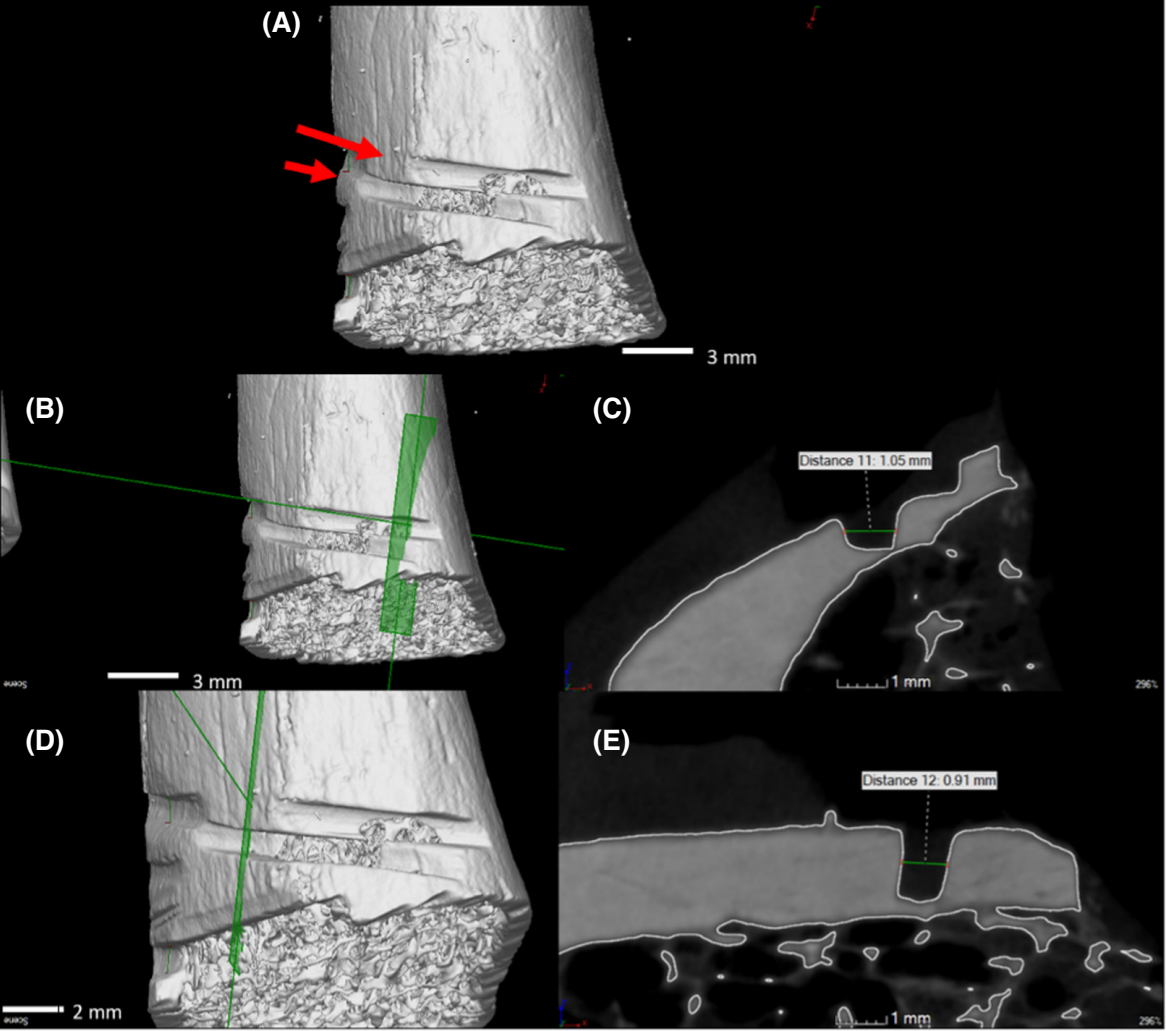
Images of false start S6.7 and S6.8; (A) medial view of the distal left radius with measurable false starts indicated, (B) cross‐sectional plane through S6.7, (C) 2D cross‐sectional profile of S6.7 showing a width of 1.05 mm, (D) cross‐sectional plane through S6.8, (E) 2D cross‐sectional profile of S6.8 showing a width of 0.91 mm

Each false start, catalogued in Figure 6, was visualized, and a 2‐dimensional cross section was produced to show the profile (Figure 2). This allowed a completely non‐destructive analysis of the false starts. For the purpose of casework, width measurements were taken from all false starts with defined walls, Figure 2, as presented by Norman and colleagues [19]. These images and measurements were provided to other experts and the forensic pathologist for final evaluation and explanation to the court. As well as profile extraction, fine details were able to be identified with the high resolution of micro‐CT including striations and light scratches on the surface of the bones (Figure 4).

**FIGURE 3:**
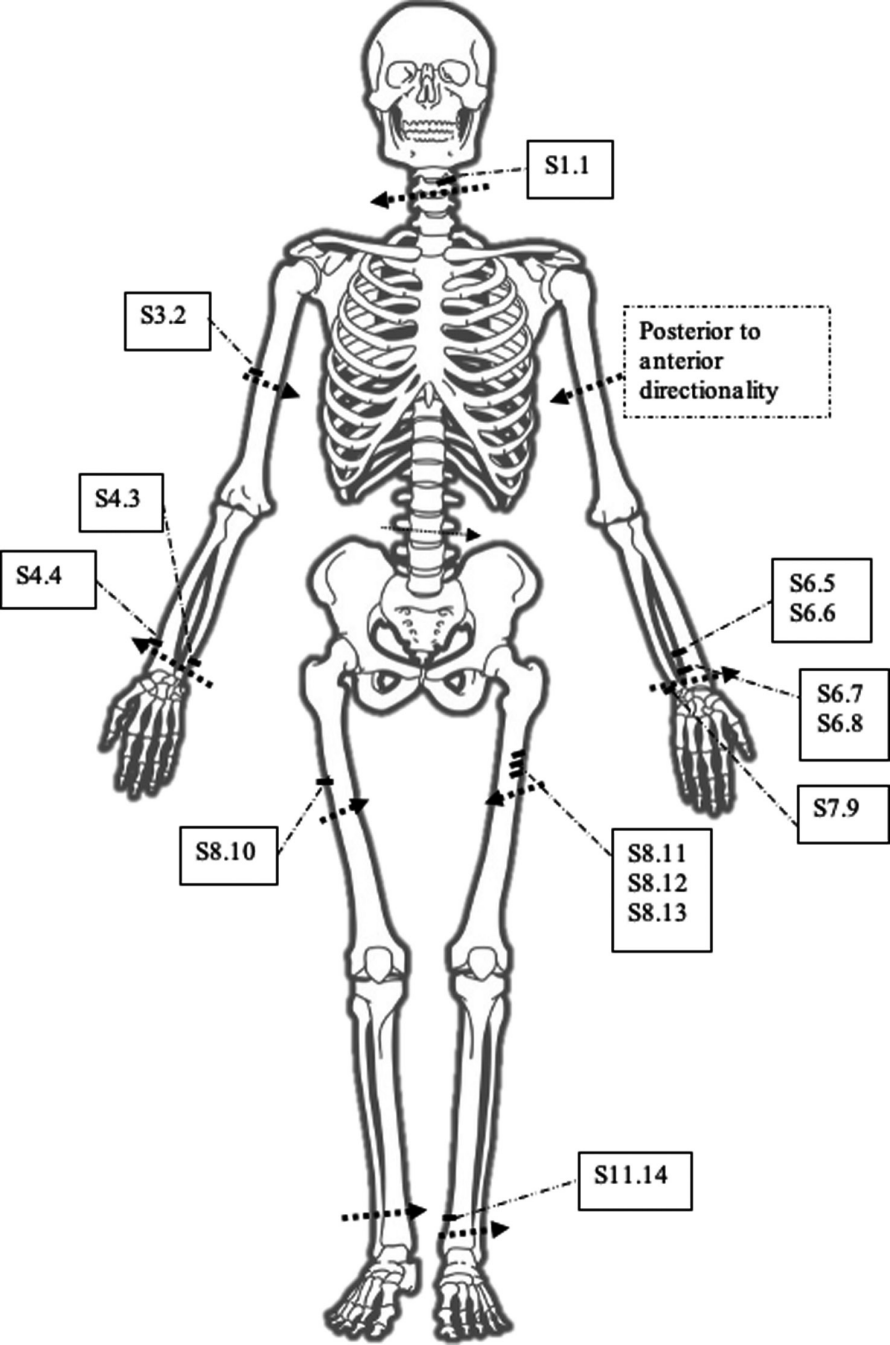
Skeletal diagram showing the approximate location and directionality of the dismemberment cuts (dashed arrows), approximate locations of the false starts, and the false start identification (boxed)

Directionality was assessed by the location of false starts and breakaway spurs and visually presented to other experts and the court (Figure 3) [28, 29]. The directionality, as disseminated by the author’s forensic report, was later correlated with that of the toolmark expert on the case, reaching the same independent conclusions.

**FIGURE 4:**
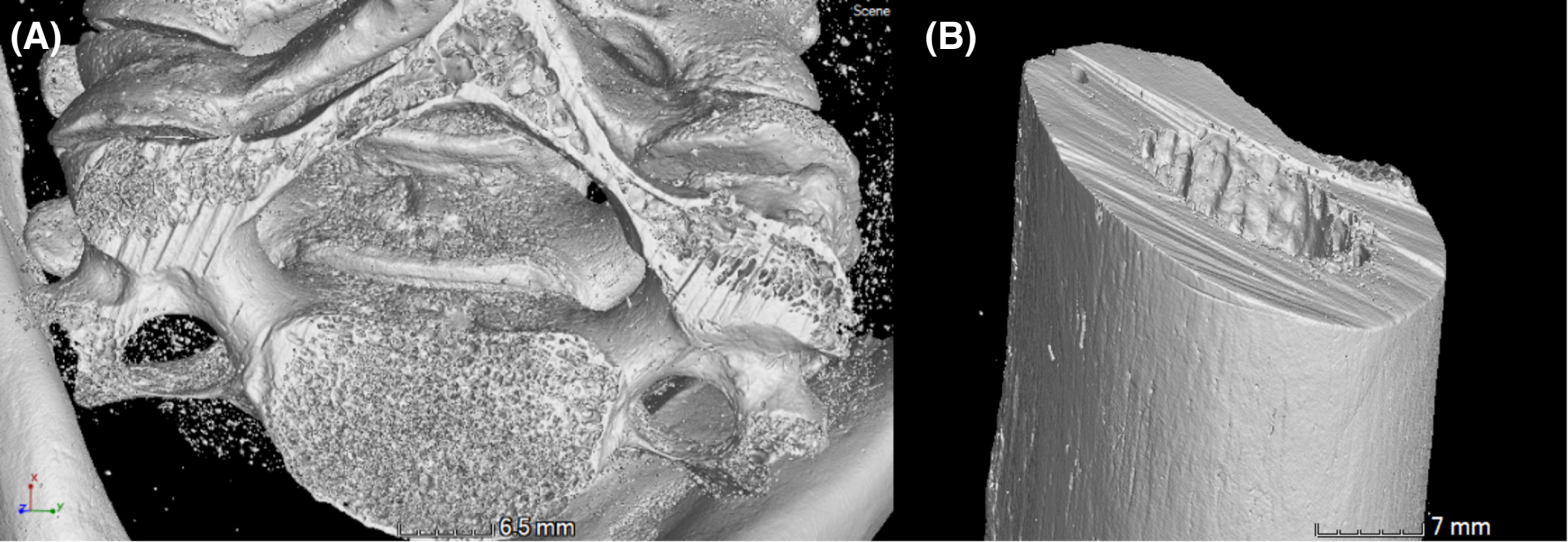
Examples of micro details visible on the digital models; (A) striations on the cut surface of the C5 on sample S1, (B) striations on the cut surface of the proximal right femur S9 and a fine scratch on the lateral surface

**FIGURE 5:**
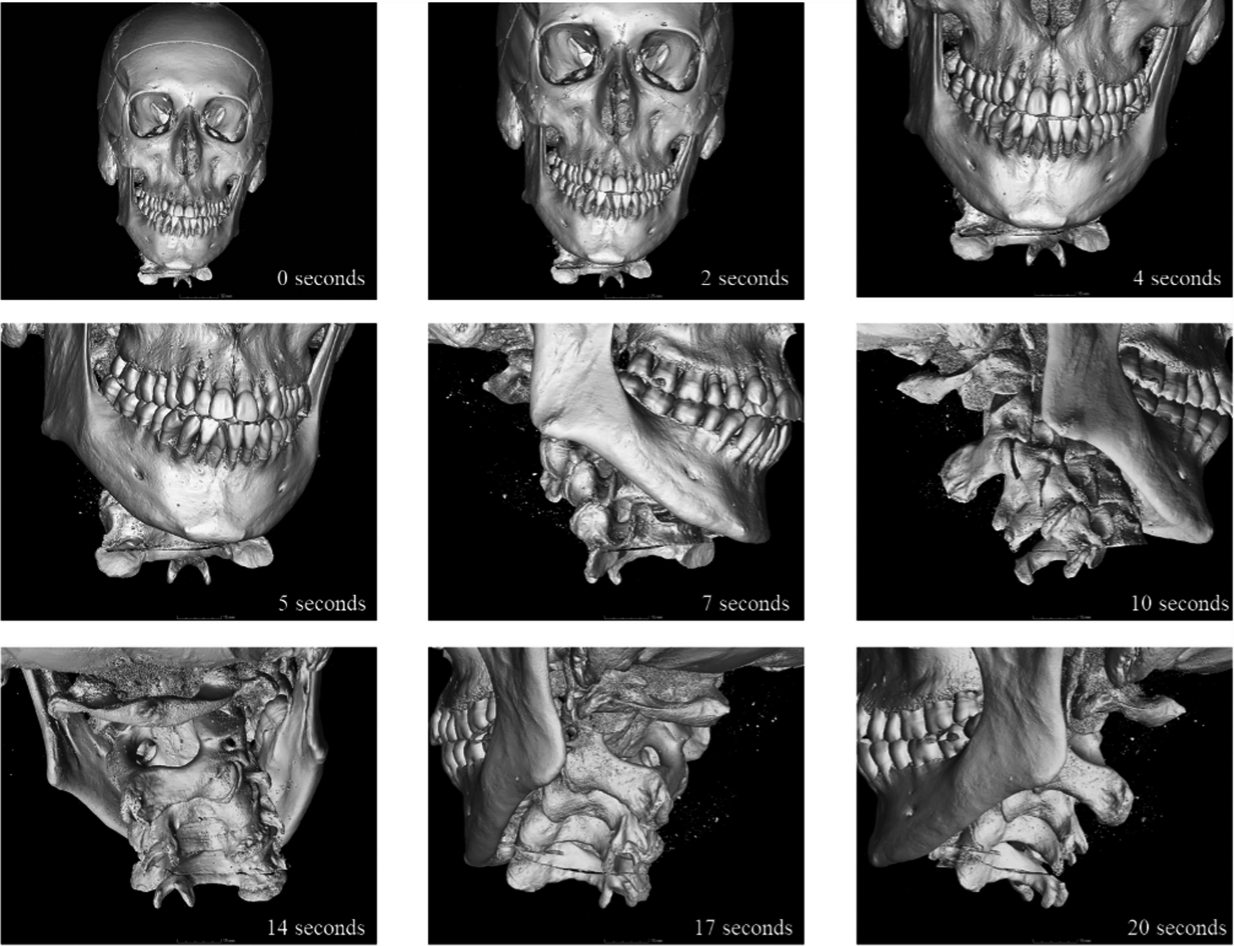
Graphical representation of the court presentation rotational video of samples S1 and S2 showing the progression of the video

**FIGURE 6:**
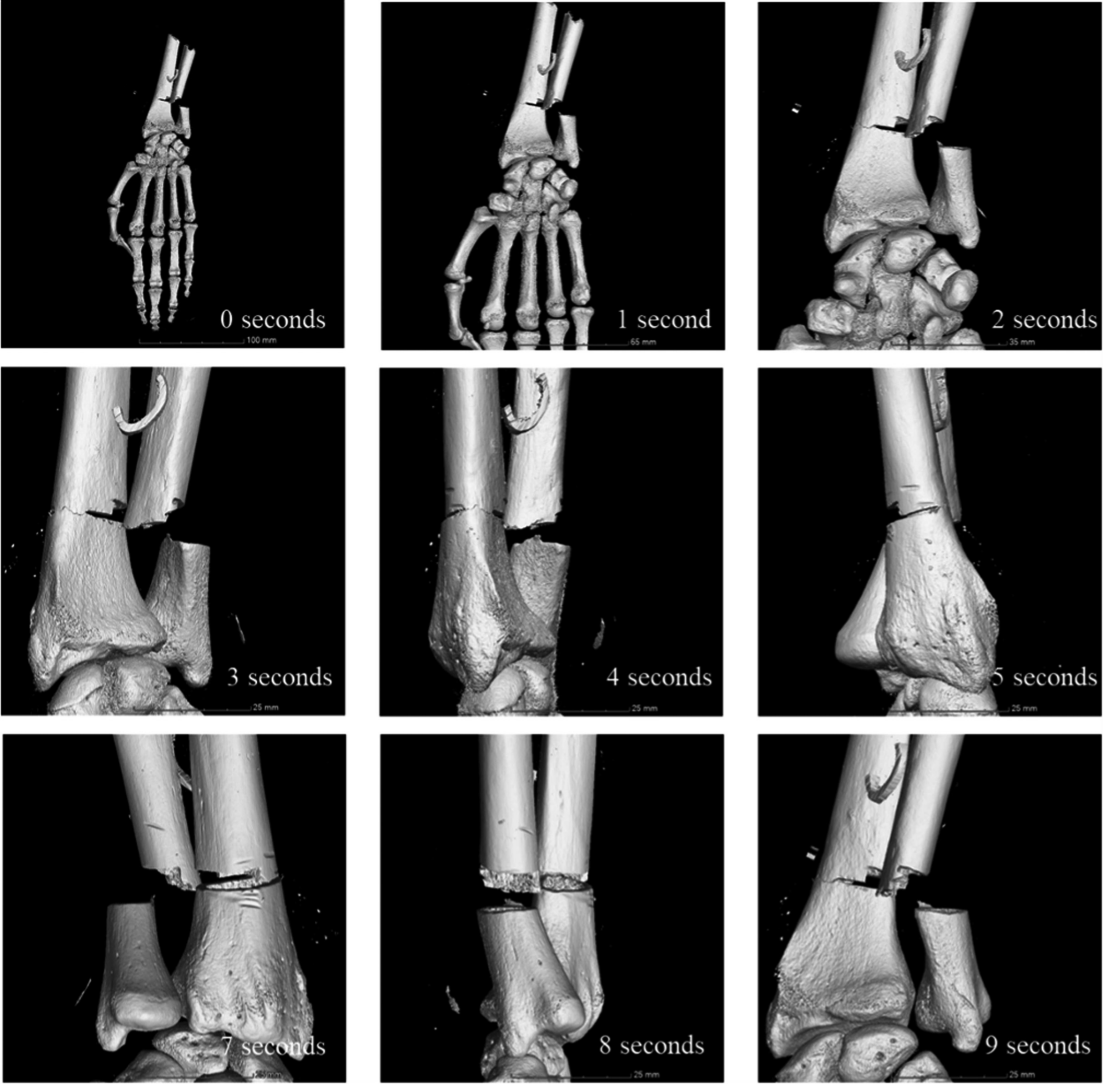
Graphical representation of the court presentation rotational video of samples S4 and S5 showing the progression of the video

Dissemination of forensic evidence in an accessible manner is a crucial element in a court case. Presentation of digital 3D models of the skeletal evidence provides a non‐distressing manner in which to convey scientific details. However, the technical capabilities of the courtroom are often limited. In this case, a slideshow was produced to display animations showing the rotation of the samples and identifying any distinguishing marks, such as false starts. The presentation aided the witness testimony of the forensic pathologist and allowed a visual understanding of the extent of the trauma to the body, and examples are shown in Figures 5, 6.

## 5 DISCUSSION/IMPACT

Implementation of micro‐CT to police casework has been previously shown to be useful to the investigative process in various types of cases including pediatric rib injuries, larynges in strangulation, and separated dismemberment [24, 25, 30]. This case review introduces micro‐CT as a high‐resolution digital alternative to the physical maceration process in a large‐scale dismemberment case.

An advantage of using X‐ray based techniques, such as micro‐CT, is the ability to distinguish between materials of different densities. In this case, packaging material and soft tissue could be digitally filtered to allow for only the skeletal matter to be visible. Digital filtration removed the need to physically macerate the samples in this case which made the investigative process more efficient, less costly, and reduced the possibility of micro‐destruction [31]. The micro‐CT scanning was completed over the course of 4 days before remains were returned to the mortuary. The estimated time for physical maceration, with transportation to an external institution and workload, had been multiple weeks, and consequently, in this case, micro‐CT examination significantly reduced the time before the remains could be reunified with the family.

Micro‐CT in this case provided a range of advantages. Firstly, the digital examination screened out any other injury not associated with the dismemberment, related to the murder, to the skeletal tissue in an efficient manner. Secondly, the need for physical maceration, which was to occur at a further external facility, was deemed unnecessary in this case, by the forensic pathologist, reducing the forensic investigation time. Finally, capturing digital models through micro‐CT enabled a preservation of the remains in a non‐invasive manner. Furthermore, the digital models are retained for research purposes in the future (with consent from the victim’s relevant family) which is essential for ongoing forensic research. Enabling a past case database for dismemberments should improve research applicability in the future.

While DNA was used to confirm the identity of the bones in this case, the matching dismembered ends of the samples provided confirmatory evidence and could, in future casework, enable unification of samples when DNA or other trace evidence is not present or possible.

Nevertheless, micro‐CT is not without its limitations. Implementation into a complex case, such as the one presented, involves 10 of hours of scanning, analysis, and court presentation production, which must be performed by a trained individual. Due to the large volume of data and specialized software, a high level of computation equipment, including secure data storage, is required. These challenges are met in UK investigations due to the close academia‐police partnership allowing application of techniques to policework where otherwise it may not be possible. Clearly, this unique relationship is not available in other jurisdictions thereby limiting widespread micro‐CT application.

Voxel resolution of <126 μm and inclusion of a calibrated workpiece enabled accurate visualization and measurement of toolmarks in this case. Measurement tools in VGStudio MAX were used to obtain width measurements on false starts. Furthermore, the location of false starts, breakaway spurs, and striations allowed the directionality of each dismemberment site to be established. False starts were catalogued to enable easy referral in relation to the sample on which they were identified and was used complementarily to the toolmark expert evidence provided externally. This allowed for a quicker investigative process and a faster reunification of the remains with the family, as noted by the Senior Investigating Officer.

The directionality, false start width measurement variability, and the extent of trauma on the body were the important narrative points when the case went to trial. The micro‐CT evaluation, in addition to forensic anthropology, toolmark, and forensic pathology expertise, provided the court with a strong case for the traumatic dismemberment. Moreover, the visualization ability of the micro‐CT scans enabled a tool to convey the large amount of evidence in an understandable way. This was achieved through witness testimony accompanying the micro‐CT report and the production of the court presentation to show, in 3D, the skeletal elements and dismemberment trauma.

This presentation improved the explanatory process of the forensic experts', particularly the pathologist’s, evidence to the jury. Visual aids may increase recognition and understanding of evidence which may also reduce explanatory time in a costly court case. The use of 3‐dimensional imagery also improves visual recognition over 2‐dimensional imaging as it is immediately recognizable and enables the jury to make a more informed decision [32]. Ensuring the evidence presentation is as non‐distressing as possible for jury members is also an important consideration. Generally, evidence must be sanitized which may reduce the conveyance of the information [33]. Presenting animations of digitally macerated skeletal elements allows for the full evidence to be presented with minimal exposure to distressing imagery, as would be seen with post‐mortem photography.

The Senior Investigating Officer and the Prosecution in this case noted the benefit of the micro‐CT report and visual court presentation provided. Remarking that the extent and complexity of the dismemberment evidence was easier to relay to the jury and enabled the variation of the false starts to be communicated. As there were multiple offenders in this case, it was important to establish whether the dismemberment could have been conducted by two individuals; while the micro‐CT evidence alone is not able to convey this, the visual representation of variation along with other evidence allowed the jury to dismiss claims that the second suspect was not involved in the dismemberment.

## 6 CONCLUSION

This report presents the evaluation and presentation of skeletal evidence in a complex large‐scale dismemberment case. The case report details the importance of employing a non‐destructive methodology to provide an option for a shorter investigative process and enhanced explanatory tools through court presentation. The two offenders were convicted of murder and will serve a life sentence.
